# Mothers’ experiences of parenting and everyday life of children born at 23 weeks of gestation – a qualitative descriptive study

**DOI:** 10.1186/s12887-020-02478-y

**Published:** 2021-01-23

**Authors:** Anniina Väliaho, Liisa Lehtonen, Anna Axelin, Riikka Korja

**Affiliations:** 1 Department of Psychology and Speech-Language Pathology, University of Turku, Turku, Finland; 2 Faculty of Medicine, University of Turku, Turku, Finland; 3 Hospital District of Southwest Finland, Department of Pediatrics, Turku University Hospital, Turku, Finland; 4 Department of Nursing Science, University of Turku, Turku, Finland

**Keywords:** Quality of life, Extremely preterm, Outcome

## Abstract

**Background:**

Surviving children born at 23 gestational weeks are a growing population. As many of these children face developmental challenges during childhood and adolescence, more knowledge is needed about the everyday life of this group. The parental perspective is important, as developmental problems often pose a challenge for the parents. The aim of this qualitative study was to explore mothers’ experiences of parenting children born at 23 gestational weeks and of the children’s everyday lives.

**Methods:**

This was a qualitative descriptive study conducted with mothers of children born at 23 weeks of gestation. These purposively sampled eight mothers were interviewed using a semi-structured interview. Thematic analysis was used to analyse the interviews.

**Results:**

Seven themes were formed on the basis of the interview data and they are presented in three dimensions: 1) *the child seen from maternal perspective* included themes ‘emphasizing strengths in the midst of challenges’, ‘relations with peers and siblings’, and ‘emotional well-being and active life’; 2) *the parenting experience* included themes ‘intensive mothering’ and ‘gratitude’; 3) *the support* included themes ‘support from the social network’ and ‘support from society’.

**Conclusions:**

The mothers described how the lives of their children were active and rich. The mothers were dedicated to motherhood and they also expressed feelings of gratitude. Mothers received support from social networks and from society. This qualitative study provided an important complementary perspective to the discussion on extremely premature children’s quality of life. It also highlighted the importance of parental perspectives in assessing neonatal care and its outcomes.

**Supplementary Information:**

The online version contains supplementary material available at 10.1186/s12887-020-02478-y.

## Background

The survival rate of children born at 23 gestational weeks already exceeds 50% in many high-income hospitals and countries [1–3]. Extremely preterm infants often have health and developmental consequences of being born preterm, and these increase the earlier the child is born [4–7]. These health and developmental problems, together with social and environmental stress factors, may impact the quality of life of families with children born extremely preterm [8]. However, parents have expressed that active neonatal care was “worth it” regardless of the outcome of their child [9].

Extremely preterm birth always includes considerable worry and stress for the parent, especially when the risk of death and developmental problems are very high, but these parental stress symptoms tend to diminish as the children grow [10–12]. Despite preterm birth-related stress, preterm infants and their mothers are as likely to form secure attachments as full-term infants and their mothers [13, 14]. Parents of preterm infants have been shown to be more protective and supportive compared to parents of full-term children [15]. The quality of parenting is one of the most important environmental predictors for the child’s quality of life [16]. In qualitative studies about parenting children with disabilities in a larger context, it has been shown that parents tend to report positive aspects about parenting children with disabilities [17].

The quality of parenting and child development in the very preterm population have received much attention in previous research literature. However, there is little long-term research providing subjective experiences of preterm infants and their parents regarding their quality of life. Jaworski et al. (2018) suggest that the lack of interest towards the parents’ perception of their child’s development could potentially lead to missing meaningful outcomes that are important to parents and families [18]. Previous studies about the quality of life of extremely preterm children have been performed using questionnaires [19–22]. The results in the questionnaire studies have varied a great deal depending on the age of the child and whether the respondent has been the parent or child. The younger the child, the more the preterm birth is perceived to affect the quality of life in a negative way. Parents have tended to report lower quality of life for their children than the children themselves [22]. A criticism of these studies has been that many quality of life questionnaires focus more on functioning in various domains and on “ill-being” rather than well-being and as such, are not consistent with the definitions of quality of life that refer to well-being [19, 23]. Qualitative research methods, especially open-ended interview questions, can provide a rich, in-depth perspective on the research topic [24] and are therefore useful in exploring the holistic concepts of quality of life and parenting.

The survival of children born at 23 gestational weeks has recently improved, but they have a high risk of developmental problems and parenting challenges. Therefore, there is a need for a deeper understanding of these children’s long-term quality of life and their parents’ experiences. The aim of this qualitative study was to explore mothers’ experiences of the everyday life and challenges of their children born at 23 weeks of gestation and the mothers’ experiences of their parental role.

## Methods

### Study approach and participants

The study approach is of a qualitative description design, which aims to describe a range of responses of a phenomenon, life event, or health and illness situation [25]. The study was carried out in a high-income country with public health care available at a low cost to all citizens. In Finland, neonatal care is guided by the family-centered care principle [26], reflecting the more general shift in neonatal care towards a more family-centered approach during recent decades [27]. The societal support in Finland includes a nine-month long parental leave during which a parent receives a maternity/paternity/parental allowance. This is complemented by childcare leave, during which the parent staying at home receives a small allowance and has a right to keep her or his job, until the child turns three years [28].

The study used purposive sampling: all families with a surviving child born at 23 gestational weeks in Turku University Hospital between 2001 and 2014 (*n* = 10) were asked to participate in the study. In this hospital, all children born at 23 gestational weeks were provided with active neonatal intensive care during the whole study period [29]. Seven families with a child born at 23^0/7^–23^6/7^ gestational weeks were willing to participate. Additionally, we included one family with a child born on 22^6/7^ gestational weeks who had received active neonatal care. Three families declined; two because of a lack of interest and one because of their busy schedule.

All participating parents signed the consent form, as well as all the children who were seven years or older. The children, born in 2003–2014, were 2, 3, 4, 8, 11, 12, 13 and 14 years old at the time the data was gathered in 2017. Six children were singletons and two had had a non-surviving twin sibling. All children had received neonatal intensive care in Turku University Hospital and multidisciplinary follow-up in the local pediatric unit until at least the age of two years of corrected age. The study was ethically approved by the Ethical Committee of the Finnish South-West Hospital District, in December 2016.

### Data collection

The data collection method was a semi-structured interview that was developed for this study (see Additional file 1). The interview consisted of six categories with pre-determined topics. Five of the categories in the interview were developed based on a thorough review of the content of questionnaires that had been used in previous studies about quality of life in children [30–34]. The categories of somatic health and functioning were adjusted with a neonatologist to include topics that were relevant to extreme prematurity (e.g. breathing, infections). The sixth category regarding the parenting experience and memories of the NICU stay was developed with the help of a psychologist specialized in the early mother-infant interaction. The interview was started with warm-up questions about the child’s age and schooling or day care situation, followed by an open question to give the parents a chance to freely describe the life of the child and the family. After that, the topics were introduced by open questions such as *“Has your child had problems [in this area]? [If yes,] what kind of problems and how they affect the life of your child and your family?”* or *“Could you please describe [this area]”.*

All interview categories are presented in Table 1. In this study, we analysed material from the interview based on our research questions about parenting and the everyday life of the child.

**Table 1:** The categories of the semi-structured, video-recorded interview of mothers of preterm infants

Open question	*“What would you like to tell me about the life of your child?”*
**1. Somatic health**	Infections, growth, pain, breathing, vision, hearing, gross motor skills, fine motor skills, continence
**2. Functioning**	Eating, sleep & vitality, daily activities, hobbies & play
**3. Learning and attention**	Learning & memory, challenges that affect learning and development, school achievement, possible special education arrangements, attention
**4. Emotional well-being**	Expression of emotions, self-regulation, anxiety, depression
**5. Social relations**	Family relations, peer relations, group skills, other important people
**6. Parenting experience**	Thoughts about how prematurity has affected child, NICU experiences, overall parenting experience, positive and negative feelings about parenting

All mothers (*n* = 8) participated in the interview. The fathers were given a possibility to participate, but only two fathers consented and therefore their interviews were not included in the analysis of the present study. One researcher (first author AV) conducted all the interviews during March to August 2017. During the process of interviewing, the first author and three senior researchers (RK, JS and LL) met regularly to watch the interview tapes; however, to establish dependability, no changes were made to the interview procedure. The interviews took place either at the home of the family or at a research facility. The length of one interview was approximately 1–1.5 h. All interviews were videotaped and later transcribed verbatim.

### Analysis of the data and authors’ positioning

We analysed the interviews using thematic analysis, which is a type of qualitative analysis to identify, analyse and report themes within the data [35]. The analysis process began in the spring of 2019, and was done by first author AV and supervised by a senior researcher (AA). The interview transcripts were read several times to obtain a holistic understanding of the mothers’ stories and the overall patterns in the data. The coding of the data was done by identifying meaning units in the text. A meaning unit in this context is considered to be words, sentences or paragraphs containing aspects related to each other through their content and context [36]. These meaning units were then labelled with codes and from the basis of these codes, twelve initial subthemes were produced. The subthemes were then further clustered into seven themes. The seven themes are presented in a three-dimensional model which provides a storyline for the identified patterns. This process is exemplified in Table 2. The research group had several comparison and validation discussions during the analysis process.

**Table 2:** Examples of meaning units, subthemes and a theme

Meaning unit	Code	Subtheme	Theme
*“I have been much more protective of her than of [the full-born sibling]. Of course, I protect [the sibling] as well, but it’s different.”*	protecting the preterm child more than the full-term sibling	maternal protectiveness	Intensive mothering
*“I haven’t even trusted [husband] to stay home to take care of our son.”*	difficulty in trusting other caregivers		
*“I have educated the coaches in her [sports team] about her special needs so that they would know how to cope with her.”*	actively participating in the child’s free-time activities	maternal dedication	
*“We were active in arranging her to start in special education. As the parent, you are the expert of your child, you know what’s good for her and what’s not.”*	advocating for the child in education decisions		

An important consideration in qualitative analysis is the authors' positioning and how it might have affected the interviewing and analysis [37]. In our research group, first author AV is an experienced clinical psychologist, which has probably been beneficial in the process of approaching the interviewees sensitively and obtaining a good contact, as this resulted in rich and detailed interview data. All members of the research group are experienced and dedicated in working with preterm children and their families, which might have caused a tendency to focus too much on the positive patterns in the data.

## Results

Seven themes were formed on the basis of the data and they are presented in three dimensions. The first dimension was *the child seen from maternal perspective* with three themes: ‘emphasizing strengths in the midst of challenges’, ‘relationships with peers and siblings’, and ‘emotional well-being and active life’. The second dimension, *the parenting experience,* included two themes: ‘intensive mothering’ and ‘gratitude’. Finally, the third dimension, *the support* that mothers and families receive, included the themes ‘support from social network’ and ‘support from society’.

### I The child seen from maternal perspective

#### Emphasizing strengths in the midst of challenges

Almost all children had one or several long-term developmental challenges or disabilities such as CP, epilepsy, severe hearing deficit, ADHD or learning difficulties, as reported by the mothers. However, all had learned the basic motor skills (walking, running, jumping) and means to communicate either by talking or sign language. Often the mothers described more specific and nuanced problems, for example, regarding motor skills, such as challenges in fine motor skills due to cerebral palsy. All school-aged children were in special education because of either learning-related or attentional problems. As learning problems manifest after the first years of life, the age of the child played a large role in mothers’ description of the children’s challenges.

*M18: During her first years of life, I didn’t notice any differences* *[between her daughter and other children].* *But when she started day care, she started to fall behind* *[compared to others].*

The developmental difficulties were different in each child and the stories varied accordingly. The mothers talked about their acceptance of the difficulties as a part of the life of their child, describing their child in a loving and accepting way. The mothers wanted to emphasize the strengths and skills of their children rather than their challenges. They also brought up milestones that the children had met age-appropriately.

*M6 (talking about her daughter who has severe learning disabilities): Yet, her motor skills have developed [age-appropriately]. When she was very young, she learnt to ride her bicycle without training wheels. And if we [parents] didn’t watch out, she would climb the ladder all the way up to the roof. (laughs).*

The children’s perseverance and resiliency were mentioned as positive personality traits. Some mothers attributed these traits to prematurity. They described how their tiny premature baby had needed the will to fight for his or her life and, later in life, the child needed perseverance to master the skills he or she had to struggle with.

#### Relations with peers and siblings

All mothers felt that their child was interested in contact with other children. For the younger children (aged 2, 3 and 4 at the time of the interview), the peer relationships naturally played a minor role, but even mothers of young children reported their children enjoying playing with others.

The mothers of school-aged children also described their children being interested in other children’s company, but a common theme was the necessity of parental help in maintaining friendships. Many children studied in special education schools that were not located near their homes. Because their peers from school lived in different parts of the town, parental effort was needed for the children to be able to visit friends outside of school-time.

*M12: (talking about her child’s friend who lived some way away from their house): For [my child], it is too demanding to go there by foot, because she doesn’t know how to be careful in traffic. But I am a friend with this child’s mother, so we always try to make it work by driving the kids and arranging meetings.*

One finding of this study was the limited number of friends the school-aged children had, even though all of them had at least one good friend. The problems in forming friendships could be a part of the child’s developmental challenges, such as autism spectrum disorder. One child with epilepsy was not permitted to visit friends without an adult being present, in case of a seizure. However, the mothers expressed their satisfaction with the children having made friends and seemed dedicated to supporting these friendships. In general, social relationships were described as an area of life that gave the children joy and strength.

Mothers often mentioned the role of the siblings when describing the children’s social relationships. As far as we know, all siblings had been born full-term and had no developmental disabilities. For the younger children, the sibling was often their most important playmate. The mothers of school-aged children described the sibling as being an important role model and a supporting figure for the prematurely-born child at school or in other social situations. Some mothers even felt that their child would not be able to do all that he or she now did, without the help of siblings.

M18: *I think [the child and her sister] have always been very close. She leans a lot on [her sister]. At school, whenever she has some kind of trouble she’ll seek out [her sister] during the breaks. It has perhaps been [my child’s] rescue that she’s had the security her big sister’s presence brings.*

#### Emotional well-being and active life

Despite the developmental challenges, disabilities, and limitations in peer relationships, a common theme in the mothers’ narratives was the descriptions of their children doing emotionally well. Mothers described their children with adjectives such as happy, joyful, or energetic. Emotional closeness between family members was often mentioned.

M36: *He is a happy child. He comes to hug and kiss us a lot. He doesn’t yet know how to say I love you, but he shows that he cares in his own way.*

Young children, as well as school-aged children, were described doing emotionally well. Mothers described some age-appropriate emotional problems, such as temper tantrums or teen-age related moodiness. However, when the mothers were asked about anxiety and depression, none of them reported their children suffered from these conditions.

The mothers described the children as having active and rich lives. All school-aged children attended school, whereas the younger children were either in a kindergarten or in the care of a day-time caregiver or a relative. The mothers generally described their children enjoying school or day care. Additionally, all the school-aged children had at least one free-time activity, such as playing an instrument, singing in a choir, scouting, or doing sports, and many did other free-time activities together with the family or with other relatives, such as their grandparents. The children’s eagerness to try and learn new things was also mentioned in some mothers’ stories.

M25: *One of her biggest strengths is having a strong self-esteem. […]* *I have no doubt that she will be able to do whatever she wants in her life.*

### II The parenting experience

#### “Intensive mothering” – dedication and protectiveness

The mothers seemed to have great dedication and commitment to parenting. They were very persistent in helping their children to thrive despite the children’s developmental challenges, and invested considerable time and effort into this. This mothering style could be described as “intensive mothering”. Many mothers had taken an active part in planning the best possible school arrangements for their child, and they also had a large role in helping their children with homework, both for learning-related and attentional-related issues. Similarly, the mothers were active participants in the children’s therapies and rehabilitation, in order to obtain the best possible outcome.

M36*: We did those exercises* *[recommended by the physical therapist] every week, and we obtained good results. I think it has really benefitted* *[my son’s] development, that all possible challenges have been tackled early on*.

Perhaps as a part of this dedicated attitude, some mothers admitted being quite protective of their children, and attributed this to stem from the child’s background as an extremely small premature baby; in the beginning the child had been so delicate and the parents had had to be very precise with their care. Some mothers described having problems in trusting other people to take care of their child, even long after the baby period.

M33*: Mostly it’s been me who has taken care of [our son]. I haven’t even been able to trust [her husband, the child’s father] enough to ask him to stay at home to care for him. [I have] some kind of a protective instinct, quite strong … at times I feel it’s perhaps even a bit too strong. I think it’s because the beginning was so tough.*

#### Gratitude

One of the motivating factors behind the mothers’ dedication and protectiveness could be the feelings of gratitude that they felt about the child having survived the difficult beginning of their life. The mothers expressed a great deal of gratitude about the neonatal care the child had received in the beginning. In general, the mothers spoke highly of the staff in the NICU.

M33: *I have such warm memories from the neonatal unit, they took care of [our son], but also of the family. […] And I think it’s incredible how they are able to treat so small [preemies].*

The gratitude was not only limited to the joy of the child having survived. A recurring theme in the interviews was an emphasis on the fact that the child’s developmental delays were, in the parent’s opinion, not that important from an overall perspective. The mothers stressed that they were grateful for the child just as he or she was.

The mothers often experienced that the very challenging NICU period had, after all, resulted in uniting the family. Some mothers felt that the child had a closer relationship with either the mother or the father, and that this closeness might be attributed to the way the parent had cared for the child in the NICU.

M25: *Her father is probably the most important thing in the world to [the child]. […] They have a very special bond. I don’t know if it might be because of that [the father] had her so much in kangaroo care in the NICU.*

### III The support system

#### Support from the social network

Despite the mothers’ intensive and dedicated parenting style, they talked surprisingly little about being tired. Often the mothers had a good social network that helped them cope. The mothers’ narratives included examples of how fathers had an active role in childcare and how marital satisfaction supported them. A larger social support system was also a recurrent theme in most interviews. Grandparents or other relatives were often related as having an active role in the everyday life of the family.

The mothers who did express feelings of tiredness usually had a quite young child with sleeping problems, and the feelings of tiredness were tolerated as the mothers believed that sleeping problems would resolve by time. Tiredness or exhaustion could also be related to a difficult life situation, not connected to the child. However, one single mother with little support described prolonged tiredness and attributed it to the child’s demanding needs.

M12: *I know that I have done my best [in parenting her child], especially considering that I have done everything on my own. […] But being a mother of a child with special needs is tiresome in general. Even organizing the everyday life is more demanding than it would be with a normal child, and then you must face the bureaucracy with schools and [different institutions providing support]. That is exhausting and time-consuming.*

#### Support from society

Aside from the family and social networks, the mothers had received support from both health care and education systems. This support played a significant role in their narratives. All children had received close developmental follow-up after their NICU period. Many mothers expressed their satisfaction with this follow-up system. Because of frequent follow-up visits, the developmental challenges had been discovered early, and the children had had good access to therapies. The mothers described therapies and special school arrangements as an integral part of their life, and mostly, with a very positive tone. Likewise, the mothers were usually satisfied with the children’s school arrangements. Many children were studying in a smaller group and had individualized learning plans. The mothers generally experienced special education arrangements as a benefit for their child and family.

*M14: He’s a slow learner, he needs more time and tuition [than the other kids]. And he can easily engage in his own thoughts during his classes. So the decision [about special education] has been good.*

A few mothers talked about rehabilitation or education arrangements that had, despite good intentions, not benefitted the well-being of the child or family. In cases when therapy appointments were too frequent or the distance to rehabilitation was too long, the burden of these support services exceeded the benefits.

*M6: She was recommended to start in a special day care facility … that was in [another town]. So it was one-hour-drive in the morning and another in the evening. She was so tired that she started to have epileptic seizures twice a month as a result*.

Despite these experiences, the overall experience the mothers reported about the support from both health care and educational system seemed very positive. This support might have played a role in helping these mothers to cope in a demanding family situation.

## Discussion

We found seven themes in this qualitative study, in which eight mothers described their experiences of parenting and the quality of life of their children born at 23 weeks of gestation. The themes were classified into three dimensions: the child seen from maternal perspective, the parenting experience, and the support. As is known from previous literature, children born at 23 weeks of gestation have a very high risk for later developmental problems [4–7]. In this study, most of the children had at least one diagnosis or disability that affected their learning or development. The mothers of the present study openly described the challenges of their children, but at the same time they emphasized the children’s strengths and skills, describing the children in a loving and accepting way. One of the challenges the children faced concerned maintaining peer relationships. Although the mothers described their children as being interested in the company of other children, the peer relationships of the children were often reported as limited, and parental help was needed in maintaining friendships. This finding seems to be in line with previous quantitative studies, as a systematic review of the social competence of very preterm born children found that children born very preterm had poorer social competence than full-term children [38]. Most mothers in this study described their child as happy and energetic. In another study about parental perspectives about young children born preterm, the main positive themes also included the child’s personality and happiness, even in circumstances where the child had a neurological impairment [18].

The mothers’ stories showed that they try to see the children’s challenges from the perspective of the child being a survivor of an extremely preterm birth. Witnessing their child survive at 23 weeks of gestation – an experience described by some mothers as “miraculous” – might have affected the mothers’ perceptions of their child in a way that later developmental problems are not seen as significant in the broader view [18, 39]. This finding seems to be in line with the results of Wraight et al. (2015), who found that mothers of preterm children, even when their child’s prognosis was not good, still remained optimistic and expressed the opinion that the active neonatal care was ‘worth it’ [9]. The mothers’ descriptions of their children’s lives provide an important complementary perspective to the discussion of quality of life. Whereas studies using quality of life questionnaires or other quantitative methodology have been criticised for focusing too much on the problems and limitations caused by developmental disabilities [19, 23], these mothers’ stories highlight that even in the midst of challenges, they still see the many strengths and capabilities of the child and their everyday life can be perceived as active and rich.

The mothers’ narratives of parenting gave a picture of an intensive, dedicated, and emotionally involved parenting style. The mothers put considerable effort into helping their children thrive, and this dedicated mothering has probably benefitted the children’s development; some mothers even expressed thoughts about perhaps having been too protective of their child. Protectiveness and supportiveness have been shown to be common features in parenting of a premature child in earlier studies [15].

In our study, the mothers’ narratives about parenting were mainly positive, although even feelings of tiredness were reported. This finding seems to be in line with a previous quantitative study about risk factors for longitudinal parental stress, in which mothers of high-risk very-low-birth-weight  children expressed more child-related stress than term mothers, but they also expressed highest levels of parenting satisfaction at 14 years [12]. Also, in qualitative studies about parenting children with disabilities in a larger context, parents have reported many positive changes in their lives as a result of parenting a child with disability [17]. It is possible that there might be some selection bias, as parents who agree for interviews might be more likely to have had a positive parenting experience. Parents’ emotional bonding to their child might make them emphasize the positive aspects of the child with disabilities.

A common theme was the support the mothers received from either their spouses and/or other family members, such as grandparents. The support from family and close friends might have helped the mothers to cope. The mothers also talked about support on a societal level, expressing their satisfaction with the close follow-up the children received after NICU, the low threshold to starting rehabilitation, and the possibility for close collaboration with hospital staff and therapists. A well-working follow-up system might be relevant to the overall well-being of preterm-born children and their families. A noteworthy factor in interpreting the results is that in Finland, most children’s therapies and rehabilitation activities are free of charge for families. Primary education is also free, and day care costs are low compared to many other countries.

This study provides a more holistic and detailed perspective about the quality of life of children born extremely preterm than previous studies using questionnaires. It also provides new and important information about the growing population of children who have survived birth at 23 weeks of gestation. We obtained rich and detailed data as the interviewer was an experienced clinical psychologist with the skills to create good contact and trust with the mothers, making it possible to cover sensitive topics.

Our study has some limitations. First, it is worth noting that the results of this study are transferable only to settings in which babies born at 23 gestational weeks can survive as a result of high-quality neonatal care. Also, we interviewed the mothers, and not the children themselves, about the children’s everyday life. Therefore, our findings may reflect more the quality of life among mothers than among the children. Although the maternal perspective and experience are important as they were the primary caretakers of children, a reporting bias needs to be taken into account when considering these findings. Another limitation was the lack of the fathers’ perspective. In Finland, fathers often participate actively in the care of their children and therefore their perspective about the everyday life of their children would be of great importance. Despite the possibility to participate, only two fathers agreed with the interview, and as a result the fathers’ interview data were not saturated enough. There might also be limitations in the range of socio-economical contexts in the data. Quantitative research shows that Finnish families of very preterm infants are usually coping reasonably well psychosocially and socio-economically [40]. Therefore, this sample does not represent a wide range of socio-economical or psychosocial contexts. As all the research group members were dedicated to and experienced in working with preterm children and their families, one can speculate whether this might have caused a tendency to focus too much on the positive patterns in the data.

A limitation concerning the method used was the structure of the interview. A less structured interview could have perhaps provided more elaborated and in-depth stories. It could even have enhanced credibility if the mothers’ stories had emerged as the mothers choose, instead of steering them with deductive questions. On the other hand, a comprehensive interview was our way to ascertain that all the relevant aspects were covered.

## Conclusions

This qualitative study was comprised of mothers of children born at 23 weeks of gestation and interviews with them about parenting and the everyday lives of their children. The mothers’ narratives of their children’s everyday lives, aside from describing the developmental challenges of the children, also emphasized the strengths of the children and their active everyday life. The parenting style of the mothers seemed dedicated and protective, and gratitude for the child was a common theme. The mothers’ stories included numerous mentions about the amount of support they received from both their family and friends and society. It is important to listen to parents’ experiences, after challenging neonatal care, to get a broad view of the quality of life of children born extremely preterm. Parental perspectives should be central in guiding neonatal care.

## Supplementary Information

**Additional file 1.** Research Interview, the interview developed for the study.

## Data Availability

None of the data used in this article is available due to individual privacy and the sensitive nature of the interviews.
