# Supplementary material for: Mothers’ experiences of parenting and everyday life of children born at 23 weeks of gestation – a qualitative descriptive study
Source: BMC Pediatr. 2021 Jan 23;21:48. doi: 10.1186/s12887-020-02478-y (PMC7825219; doi:10.1186/s12887-020-02478-y)
Supplement: Supplementary file 1 — Additional file 1. Research Interview, the interview developed for the study. [file 12887_2020_2478_MOESM1_ESM.docx]

**Research interview**

*Most of the interview questions were presented to all participants. The questions separated by a margin are defining questions, which were presented to participants in need of help to elaborate the theme in question. If the interviewee spontaneously gave an elaborate answer that included the same information, the defining question wasn’t presented.*

**Warm-up questions.**

*(Welcoming the parent, telling about the video and audio recording.)*

How old is your child at the moment?

*(For the parents of school-aged children):* What class does your child attend at the moment? What kind of a school does (s)he attend?

*(For the parents of children under school age):* Does your child currently attend day care or kindergarten, or are you taking care of him/her at home?

*(If the child is in day care):*

When did your child start attending day care?

This interview will be started off with an open question. What would you like to tell me about the life of your child and his / her life, when you think about it from the very beginning until this moment?

*(The parent is let to freely describe the life of his / her child, and the researcher doesn’t ask defining questions during this part of the interview.)*

*(If the parent has trouble getting started, a prompting question will be asked):*

You can start, for example, by describing the first years of your child.

**Introduction to theme questions.**

I will now ask you questions about different themes that have to do with quality of life. As this is a research interview, all participants will be asked the same questions. All questions might not feel very well suited to the situation of your particular family. In spite of this, please answer to each question as well as you are able.

**Theme 1. Somatic health.**

**General health**

Could you please describe your child’s health in general?

How often does your child approximately get sick, for example, with flu or with fever?

Do you find your child is sick more often than his peers or siblings?

**Growth, body image**

Does your child grow according to the growth expectations?

*(If not):*

Could you describe the problems with growth?

Does it seem to you that your child is content with the way he looks, with his length and his weight?

**Pain**

Does your child suffer from some kind of physical pain or ache – e.g. stomach ache or headache?

*(If yes):*

Can you describe these pains and their regularity?

Do you find your child suffers more of these pains than his peers or siblings?

How do these pains affect the life of your child and your family?

**Breathing**

Is your child able to breathe easily, or does he have asthma or asthmatic symptoms?

*(If the child suffers from asthmatic symptoms):*

How do you find these symptoms affect the life of your child and your family?

Does your child have medication for asthma?

*If yes:*

Is the medication used regularly? How often?

**Vision**

The next question is about vision. Does your child have normal vision, or does he have to wear glasses in order to be able to see clearly?

*(If the child wears glasses or has problems with vision):*

How do you find that using glasses has affected the life of your child?

Does it affect the place (s)he has in the classroom?

Does it affect his/her choice of hobbies?

**Hearing**

Does your child have normal hearing, or does (s)he have to use some kind of a device to be able to hear clearly?

*(If the child has a hearing device):*

How do you find that wearing the device has affected the life of your child?

**Gross motor skills**

The next question is about motor skills. When you think of your child with regard to his peers, do you find that your child’s basic motor skills, such as walking, running and jumping, are equal to those of his/her peers?

*(If the child has problems with motor skills):*

How do you find these problems have affected the life of your child and your family?

Has your child already learned / at what age did your child learn to a) walk, b) drive a bicycle without training wheels, c) swim?

If there has been some trouble learning these skills, could you please describe these problems?

**Fine motor skills**

Again, please think of your child with regard to his peers. How do you find your child does with activities that require fine motor skills or prestidigitation? For example, to tie his shoelaces, cut with scissors, eat with a knife and a fork?

(*If the child has problems in this area):*

How do you find these problems have affected the life or your child and your family?

**Continence**

Next, some questions about your child’s continence.

Is your child able to use the bathroom on his own?

Does (s)he need to be reminded of bathroom visits?

Has your child had trouble with bedwetting and/or daily wetting himself?

*(If the child has problems in this area):*

Does the child use diapers?

How do you find these problems have affected the life of your child and your family?

**Theme 2. Functioning**

**Eating**

How would you describe your child’s eating behaviour?

Does (s)he eat independently?

*(If the child does not eat independently):*

Could you please describe the type of help your child needs?

Does your child have a good appetite?

Some kids tend to be picky with what they want to eat and what they don’t. Is your child picky in this way?

*(If yes):*

Could you elaborate on this? What do you think this pickiness originates from?

How do you think eating problems affect the everyday life of your child and family?

**Sleep, vitality**

How would you describe your child’s sleeping behaviour?

*(If the following hasn’t come up already):*

Does (s)he have trouble falling asleep? What about sleeping through the night?

All children have varying days when it comes to feeling energetic throughout the day. How do you think your child is most of the time – does it seem to you that (s)he is energetic and has enough energy to cope with the activities of the day?

*(If not):*

Do you find your child struggles with a lack of energy?

Do you find that your child would seem more tired or lacking energy than his siblings/peers?

How do you find the problems with sleep and/or vitality affect the life of your child and family?

**Daily activities**

Again, please think of your child with regard to his/her peers. How do you find your child does with daily activities such as getting dressed, taking a shower, brushing his teeth, etc.?

*(If your child has problems in this area):*

Could you please describe these problems?

How do you find these problems affect the life of your child and family?

**To parents with kids under 13: Play**

Could you describe the way your child likes to play?

Is there something in your child’s play skills that either you or your child’s teacher have worried about?

*(If yes):*

How do you find these problems affect the life of your child and your family?

**Hobbies and free-time activities**

What kind of hobbies or free-time activities does your child have?

*(If the child has some kind of a hobby or a free-time activity):*

How often does your child go to [this activity]?

Is there something with your child’s hobbies that you or the instructor would have been worried about?

*(If the child doesn’t have any free-time activities):*

Could you describe why not?

**Screen time**

How much time screen time does your child have, e.g. watching television, using a tablet, a cell phone or a computer?

Do you know what your child does with a computer or a tablet?

Do you think your child has too much screen time?

*(If yes):*

Do you find this is notable in your child’s behaviour? In what way?

Is there some kind of application or a digital program that helps your child in some area of his development?

**Theme 3. Learning and attention**

**Learning & memory**

Could you please describe how you find your child as a learner of new things?

What about his memory, how well do you think your child remembers the things (s)he’s already learned?

Is there anything about the learning skills of your child that you or the child’s teacher have been worried about?

*(If yes):*

Could you please describe these problems? How do you find these problems have affected the life of your child and your family?

**Academic achievement**

Could you please describe your child’s achievement at school / kindergarten / day care?

Does your child like school / kindergarten / day care?

Why do you think (s)he likes it / Why do you think (s)he doesn’t like it?

What school subjects / day care activities does he like?

Is there any subject or activity your child has had problems learning?

*(If yes):*

Could you please describe these problems? Why do you think this has been difficult for your child?

Does your child have specific learning arrangements due to learning challenges?

*(If yes):*

Could you describe these arrangements and the way affect the life of your child and your family?

**Attention / concentration**

How would you describe your child’s ability to be attentive and to concentrate on different kinds of activities?

*(If the child has trouble concentrating):*

Could you please describe what kind of situations do these troubles arise?

How do you find these troubles affect the life of your child and family?

Are there activities in which your child seems to be able to concentrate for a longer time period?

**Theme 4. Emotional well-being.**

**Emotions and self-regulation**

Could you please describe how you find that your child feels and shows different emotions, such as sadness, happiness, anger?

How do you find your child is able to regulate his/her feelings?

How does your child calm himself when (s)he’s upset?

What does he do when (s)he feels sad?

Is there anything with your child’s emotional well-being or self-regulation skills that either you or the child’s teacher have worried about?

*(If yes):*

Could you please describe these problems? How and in what kind of situations do the problems arise?

**Distress, anxiety, depression**

Do you think your child often seems distressed or anxious?

*(If yes):*

What do you think these feelings originate from? Have you discussed it with your child? How do you find this affects the everyday life of your child and your family?

Has your child ever been diagnosed with depression or anxiety?

*(If yes):*

How does this affect the everyday life of your child and family?

**Theme 5. Social relations.**

**Family relations**

Could you please describe the type of relations that your child has with family members, parents, and siblings?

Is there anything about your child’s relations with family members that have caused you worries?

*(If yes):*

Could you please describe this further?

**Relationships with friends**

*(Only to parents of children younger than the age of five):* Do you find your child is interested in other people?

Does your child have friends?

*(If yes):*

Could you please describe the relationships that your child has with his/her friends?

Could you please describe how you find your child functions in a group?

Is there anything about your child’s social relations that you or the child’s teacher have been worried about?

*(If yes):*

Could you please describe these problems?

**Other relationships**

Are there other important people in your child’s life than parents, siblings, and friends?

(If yes):

Could you tell me about your child’s relationships with these people?

**Theme six. Parenting experience.**

Your child was born extremely premature, and he needed a long neonatal intensive care period at the hospital. Today, how do you think being born prematurely has affected the life of your child?

I will now ask some questions about the neonatal intensive care period. Could you describe the neonatal intensive care period? How did you feel during that time?

Are there any particular events that you still remember from the NICU?

How would you describe the development of the bond between you and your child in the beginning? What was helpful to you in bonding with your child?

At what phase did you start to have confidence that your child would survive?

Could you please describe the memories you have about the first days and weeks of having your child at home?

Now, I am asking you to think about the current situation again. Could you please describe the way you feel about being the mother / father of your child?

What brings joy to you about parenting your child?

What are the challenges or stressors you find in parenting your child?

**Final.**

I have now asked all the questions that are presented to each participant. You are now able to again openly describe the life of your child in your own words.

If you find something important was left out in any of the themes we discussed, feel free to elaborate that theme now.

*Turning off the video and audio recording, thanking the parent.*
